# Bakta Web – rapid and standardized genome annotation on scalable infrastructures

**DOI:** 10.1093/nar/gkaf335

**Published:** 2025-04-24

**Authors:** Sebastian Beyvers, Lukas Jelonek, Alexander Goesmann, Oliver Schwengers

**Affiliations:** Bioinformatics and Systems Biology, Justus Liebig University Giessen, Ludwigsplatz 13-15, 35390 Giessen, Hesse, Germany; Bioinformatics and Systems Biology, Justus Liebig University Giessen, Ludwigsplatz 13-15, 35390 Giessen, Hesse, Germany; Bioinformatics and Systems Biology, Justus Liebig University Giessen, Ludwigsplatz 13-15, 35390 Giessen, Hesse, Germany; Bioinformatics and Systems Biology, Justus Liebig University Giessen, Ludwigsplatz 13-15, 35390 Giessen, Hesse, Germany

## Abstract

The Bakta command line application is widely used and one of the most established tools for bacterial genome annotation. It balances comprehensive annotation with computational efficiency via alignment-free sequence identifications. However, the usage of command line software tools and the interpretation of result files in various formats might be challenging and pose technical barriers. Here, we present the recent updates on the Bakta web server, a user-friendly web interface for conducting and visualizing annotations using Bakta without requiring command line expertise or local computing resources. Key features include interactive visualizations through circular genome plots, linear genome browsers, and searchable data tables facilitating the interpretation of complex annotation results. The web server generates standard bioinformatics outputs (GFF3, GenBank, EMBL) and annotates diverse genomic features, including coding sequences, non-coding RNAs, small open reading frames (sORFs), and many more. The development of an auto-scaling cloud-native architecture and improved database integration led to substantially faster processing times and higher throughputs. The system supports FAIR principles via extensive cross-reference links to external databases, including RefSeq, UniRef, and Gene Ontology. Also, novel features have been implemented to foster sharing and collaborative interpretation of results. The web server is freely available at https://bakta.computational.bio.

## Graphical Abstract

**Graphical Abstract:**
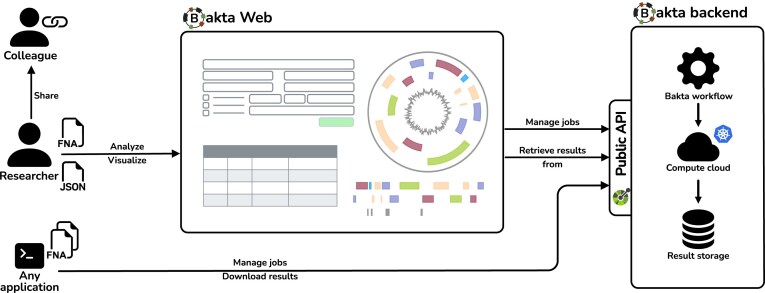

## Introduction

Genome annotation is a crucial step in microbial whole-genome sequencing analysis, immediately impacting numerous downstream analyses. Various well-established command line tools, such as Prokka [1], PGAP [2], and DFAST [3] were developed following different approaches, each offering distinct trade-offs between comprehensive annotations and computational demands. In 2021, Bakta [4] was introduced as an additional alternative, leveraging an alignment-free sequence identification (AFSI) approach to provide a more-balanced solution between speed and accuracy.

Since its release, Bakta has been widely adopted by the research community, benefiting from extensive community-driven improvements, including bug reports, feature requests, and code contributions. It has become a well-established tool for both single-genome and large-scale annotation projects, such as BakRep [5] and AllTheBacteria [6].

However, as annotation workflows evolve and database sizes grow, computational demands have increased, posing challenges for researchers with limited access to required resources. In addition, sufficient command line expertise might pose an undesired technical barrier for some users. To address these issues, an accompanying web server for Bakta was developed, offering a user-friendly alternative. Since its launch, the web version has gained significant attraction, with >15 700 bacterial genomes annotated in 2024 and a continuously increasing annotation rate of >2500 genomes per month.

In response to these demands, the Bakta web server has undergone major enhancements, including improved scalability, reduced runtimes, and novel features such as an interactive circular genome plot. Here, we present the improved Bakta web server, describing its expanded capabilities, backend re-implementation, and integration with the latest Bakta CLI updates.

## Materials and methods

The Bakta Web frontend is written in TypeScript using the Vue.js framework v3.5.12 (https://vuejs.org) and Bootstrap v5.0.0 (https://getbootstrap.com). Web visualizations are implemented in d3.js v7.9.0 (https://d3js.org), and the interactive genome browser is based on the Integrated Genome Viewer (IGV) igv.js v2.8.3 [7]. The backend is written in Rust using the tokio v1.43.0 async stack (https://tokio.rs), Axum v0.8.2 (https://github.com/tokio-rs/axum) as a web server, and Utoipa v5.3.1 (https://github.com/juhaku/utoipa) for OpenAPI generation. Both, backend and frontend are deployed as containerized applications on a Kubernetes (https://kubernetes.io) cluster with job scheduling managed via Argo Workflows (https://argoproj.github.io/workflows), a Kubernetes-native workflow engine. The compute cluster dynamically scales from three nodes with a total of 84 CPU cores to a current maximum of 12 nodes with a total of 336 cores. All annotation jobs utilize the standard unmodified Bakta Docker image available at DockerHub (https://hub.docker.com/r/oschwengers/bakta), ensuring consistency between web-based and command line annotations.

## Description of the webserver

The Bakta web server is a convenient service providing access to the full feature potential of the Bakta command line version. Thus, researchers with limited command line experience or lacking access to sufficient hardware capacities find a more approachable interface for smaller annotation tasks or projects. It eliminates the need to download and maintain large annotation databases [8] (∼30 GB compressed, 84 GB unpacked) and regular software updates required for local Bakta installations. Furthermore, the Bakta web server offers various interactive visualizations facilitating the interpretation and comprehension of detailed annotation results.

To customize the annotation process, Bakta Web accepts various user inputs. Besides bacterial genome sequences in FASTA format, including complete and draft genomes, plasmids, and metagenome-assembled genomes, additional information is accepted, as for example genus, species, and strain designations (used for naming purposes only), minimal contig lengths, the genetic code or coding table selection, and an existing Prodigal [9] training file useful to achieve better gene prediction results on smaller sequences like plasmids. Furthermore, users can provide detailed per-sequence information via a dynamic table accepting the following information: new identifiers, replicon types, i.e. contig, chromosome, plasmid, topology, i.e. linear or circular, and a name.

The system leverages a state-of-the-art tech stack dynamically providing and orchestrating cloud computing resources to process incoming annotation tasks and thus, makes the tool accessible to researchers without required local computational infrastructures. The output generated by Bakta Web includes a suite of annotation results in standard bioinformatics formats, such as GFF3 and the INSDC formats GenBank and EMBL for the seamless interoperability with downstream analysis tools; annotated and hypothetical protein sequences in FASTA format; detailed feature tables as TSV; and a comprehensive custom JSON format containing all information obtained and used in the annotation process. Feature type-wise, the system provides annotations for coding sequences (CDSs), non-coding RNAs e.g. ribosomal RNAs (rRNAs), transfer RNAs (tRNAs), non-coding RNAs (ncRNAs), and DNA features like CRISPR arrays, origins of replications, and origins of transfers. Each annotation includes functional descriptions and cross-references to external databases including RefSeq [10], UniRef [11], and Gene Ontology [12] identifiers.

**Figure 1.**
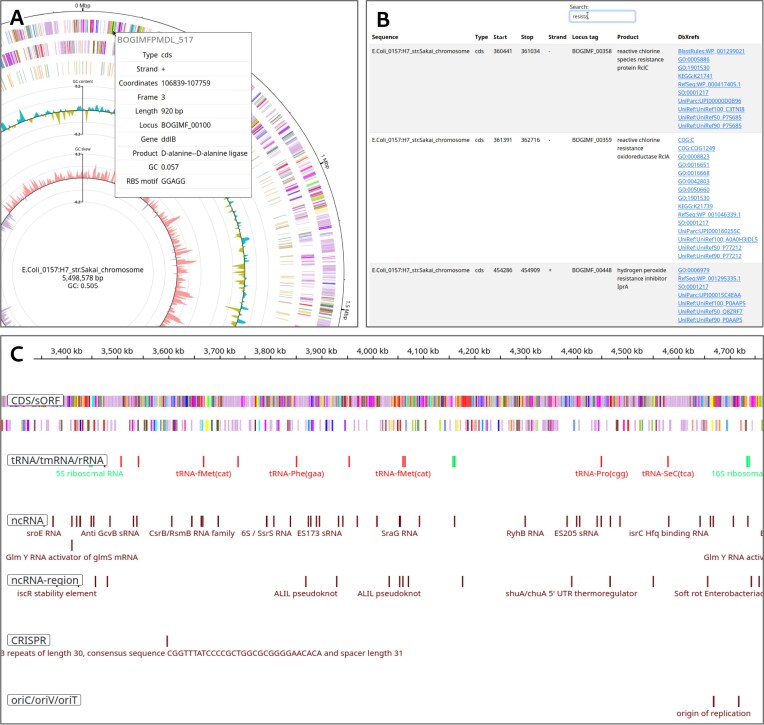
Interactive visualizations of annotation results. Presented are genome annotation results of the *Escherichia coli* O157:H7 str. Sakai chromosome. (**A**) A circular genome plot, including tracks for CDS on the forward and reverse strand, non-coding features, GC-content percent, and GC-skew from outer to inner tracks. (**B**) A searchable data table providing detailed feature information and cross-reference links. (**C**) A linear genome browser with customizable tracks for CDS, tRNAs, ncRNAs, ncRNA-regions, CRISPR regions, and oriC/oriV/oriTs based on IGV.

To assist researchers in studying the broader genomic architecture of annotated sequences, identifying regions of interest, or investigating single genes, Bakta Web offers three interactive visualization widgets providing insights into the vast annotation results on different levels of detail without the need for additional software, i.e. a circular genome plot, a linear genome browser, and a detailed data table (Fig. 1). Of note, the platform also supports the visualization of locally generated results in JSON format, thus enabling command line users to benefit from the same interactive visualization features while maintaining their preferred workflows and keeping sequences and results confidential, as all data is strictly handled in the local web browser.

### Annotation pipeline and database updates

Since its first stable public release (v1.0) and the release (v1.1) that has been described in the initial publication, Bakta’s annotation workflow has been expanded, many novel features have been developed, countless bug fixes were applied, and most internal data structures have undergone substantial re-writes and significant optimizations. Most importantly, Bakta is now able to identify and annotate CDS pseudogenes and causative pseudogenization events like point mutations and frameshifts, thus substantially improving the impact of CDS annotations. Another major update addresses translational exceptions by detecting and annotating selenocysteine-containing CDS, which are often mispredicted by gene prediction tools. To further enhance CDS annotations, Prodigal [9] was replaced by Pyrodigal [13], a modernized and actively maintained alternative fixing several bugs severely affecting *de novo* gene prediction accuracy. To increase gene symbol consistency within operons, a new gene symbol harmonization feature has been developed that automatically selects and assigns the most-appropriate gene symbol based on a gene’s neighborhood context. To allow for detailed comparisons and critical assessments of annotation confidence and quality, annotation inference metrics are now exported in a dedicated TSV file. Finally, compliance of Bakta result files with INSDC genome submission standards as well as community best practices has been improved.

In addition to these software and workflow updates, both content and structure of Bakta’s underlying mandatory taxonomy-agnostic database were regularly updated and expanded. For instance, between database releases v3.0 and v6.0, the numbers of pre-annotated protein sequence cluster of clusters, protein sequence clusters (PSCs), identical protein sequences (IPSs), and unique protein sequences significantly increased from 12.1 to 37 M, 90.6 to 135.3 M, 199.6 to 330.9 M, and 214.9 to 350.6 M, respectively. The massive number of pre-calculated protein sequence hash fingerprints allows the rapid identification of known protein sequences without costly sequence alignments for a large proportion of genomes. For example, based on a taxonomically diverse set of 49 public genomes, 92.8% of all annotated CDS (159 161 from 171 610) could be exactly identified via the AFSI approach. Besides the pure increase of known protein sequences and sequence clusters, also the functional pre-annotation of IPSs and PSCs in general has been significantly enhanced using several high-quality annotation resources, as for instance NCBI COG [14] and NCBIfams [15], KEGG [16] Kofams, and PHROGs [17]. Also, comprehensive cross-references to databases like RefSeq and KEGG, as well as functional categories, as for example, COG and Enzyme Commission numbers [18] have been applied. To store additional annotation data in a compact and efficient manner, the versioned database schema was constantly improved and adopted. However, these developments nevertheless led to substantially increased database storage requirements from 53 to 84 GB between v3.0 and v6.0 driving the demand for and usage of the Bakta web server.

### Improved web user interface

Comprehensive annotation workflows generate huge amounts of results, exported in various potentially large human- and/or machine-readable text formats. Thus, effective visualizations are of paramount importance for the comprehension, interpretation, and subsequent analysis of annotation results. Addressing this task, Bakta Web provides a comprehensive web viewer to explore generated results on various levels of detail. The updated interface either collects annotation results from the backend’s S3 cloud storage or optionally accepts local JSON result files. Results are then aggregated and organized within a structured, tab-based view, providing users with a cohesive and interactive platform for data exploration. It is divided into four distinct sections, each dedicated to provide the information from a different point of view. The first tab provides a unified job summary, presenting both technical run-time metrics and sequence annotation metrics, i.e. GC content, N50, N90, coding density, N ratio, and feature count statistics. This summary allows for quick assessments of the quality of both the genome and annotated features, as well as the computational performance of the analyzed sequences. A second tab features the novel circular genome viewer that provides general insights into the larger genomic architecture facilitating the rapid assessment of genomic composition and structural characteristics. Dedicated tracks present information on annotated features, GC content, and GC skew across individual regions (Fig. 1A). Features are colored according to available COG annotations or feature type otherwise. To investigate individual sequences and to zoom into regions of interest, annotated features down to single nucleotides resolutions, a third tab provides an interactive and dynamic linear genome browser utilizing IGV.js (Fig. 1C). The genome browser displays dedicated tracks for CRISPR sites, gaps, and various origins of replication, enabling a more precise examination of genomic regions of interest. To provide all available information in full detail, a fourth tab features a searchable data table listing all annotated features, providing convenient access to enriched annotation information via links to external databases exploiting Bakta’s comprehensive cross-references, e.g. RefSeq, UniRef, and Gene Ontology identifiers (Fig. 1B). Fostering the FAIR principles [19] by these integrations allows users to efficiently navigate through the results and to contextualize individual annotations within a broader biological framework. Of note, unique protein sequence identifiers also help to gain further insights on hypothetical proteins—now or in the future. A tiny but important enhancement are new sharing and bookmarking buttons allowing to share jobs and annotation results via a unique URL with other researchers thus, fostering collaborations.

### Faster and auto-scalable cloud backend

We completely rewrote the Bakta Web backend addressing three primary goals, i.e. reducing annotation runtimes per job, increasing scalability and total job throughput to meet increasing demands, and last but not least, achieving better code maintainability for a future-proof platform successfully coping with continued growth in bacterial genome research. In the old setup, network-attached copies of the database posed a severe performance bottleneck when multiple jobs needed to access it simultaneously, particularly the SQLite part of the database. By replacing the single network-attached central database instance by decentralized copies stored on fast SSD devices locally-attached to each compute node, we eliminated this bottleneck. Now, Bakta Web processes bacterial genome annotations up to three times faster than before, with average job completion times reduced by 70%. This change is particularly beneficial for unknown CDS that are not identified via the AFSI approach requiring extensive database searches. Hence, granted compute resources are used in a more efficient manner allowing to annotate more bacterial genomes in significantly shorter time periods. To further meet the growing annotation demands of our users, Bakta Web utilizes a new and cloud-native backend implementation leveraging a state-of-the-art tech stack that dynamically adapts the number of used compute nodes to current demands, thus allowing for a most-efficient usage of cloud computing resources provided by the de.NBI network. In times of higher demands and peak workloads, the backend automatically and rapidly expands its capacity and scales back during quieter periods, reducing its overall environmental footprint. This enhanced setup is transparent to users and it is designed to seamlessly accommodate future increases in both the number of users and jobs, as well as more complex annotation tasks, thus ensuring that Bakta Web will remain a reliable resource for bacterial genome annotation as the field continues to evolve.

### Enhanced interoperability via a modernized web API

To improve and foster the accessibility and integration capabilities of our service, we have substantially modernized the underlying Web API. This API is used by the Bakta Web frontend but also enables direct programmatic access by other services or from the command line as part of larger workflows and pipelines. To this end, the API has been migrated to OpenAPI 3.1 specifications, allowing for better documentation and generation of client libraries in various programming languages. It now supports all additional Bakta output result files and features improved job status tracking, facilitating swift and easy monitoring of annotation jobs in real-time. We have also added an endpoint to retrieve the Bakta command line standard output (stdout) from running and completed jobs. This access to detailed per-process log files helps to gain and provide useful information for general debugging purposes and troubleshooting of unexpected results, which is particularly helpful for genomes of understudied taxa.

## Discussion and conclusion

In this article, we presented Bakta Web, an improved web application for rapid and comprehensive bacterial genome annotation. Bakta Web provides convenient and up-to-date access to the well-established Bakta command line tool via an interactive web application leveraging state-of-the-art cloud computing infrastructure and scalable compute capacities. By democratizing access to high-quality genome annotation through a simple web-based interface, Bakta Web significantly reduces technical barriers for researchers who may lack command line expertise or access to sufficient computational resources. This accessibility is particularly valuable for researchers in settings with constrained infrastructure or limited technical support, enabling broader participation in bacterial genomics research across the global scientific community. The platform eliminates the need to download and maintain the substantial annotation database (∼30 GB compressed, 84 GB unpacked) required by the command line version, while still delivering the same comprehensive annotation capabilities and accuracy that have made Bakta a widely adopted tool in the microbial genomics community. The interactive visualization features integrated into Bakta Web represent a significant enhancement over the command line experience, allowing researchers to easily explore genomic features and to identify biological patterns more effectively. These visualizations are designed to complement rather than replace the command line version, offering benefits for both web-only users and experienced command line users. Notably, researchers who prefer the command line version for batch processing or pipeline integration can still leverage the web interface capabilities by visualizing locally generated Bakta results on the website without requiring any data uploads to our servers. This seamless integration between command line and web workflows provides unparalleled flexibility across different technical environments and user preferences. The platform’s cloud-based architecture ensures consistent performance regardless of local hardware limitations, while the automated scaling capabilities accommodate varying demand patterns without compromising annotation quality or speed. This infrastructure design represents a careful balance between accessibility, computational efficiency, and robust performance, making high-quality bacterial genome annotation available to a broader scientific audience. When compared to existing annotation services, Bakta Web offers several distinct advantages. Unlike RAST [20], which requires users to create and maintain registered accounts, Bakta Web provides immediate access without any registration barriers. Additionally, while PGAP is widely recognized for its high-quality annotations, it lacks a dedicated public web service and must either be run locally or is automatically executed upon NCBI genome submissions. Bakta Web bridges this gap by offering a sweet spot between annotation quality and wall clock runtimes comparable to these established tools with superior accessibility and user experience. By offering sharing and bookmarking features, Bakta Web provides a simple but effective set of collaboration features. Looking forward, we plan to extend Bakta Web’s capabilities by implementing support for user-provided inputs, including custom protein sequences, hidden Markov models, and genomic regions of interest. While these features are already available in the Bakta command line version, they are currently unsupported in the web interface. Adding these capabilities will allow web users to incorporate specialized domain knowledge into the annotation process, enabling more-targeted analyses while maintaining the platform’s user-friendly approach. This website is free and open to all users and there is no login requirement. The Bakta web server is available at: https://bakta.computational.bio

## Data Availability

The complete source code for all components is publicly available. The frontend user interface/website is available at https://github.com/ag-computational-bio/bakta-web-ui (DOI: 10.5281/zenodo.14973849) under a GPL-3.0 license. The backend implementation can be accessed through the Bakta Backend repository at https://github.com/ag-computational-bio/bakta-web-backend (DOI: 10.5281/zenodo.14973432) under a GPL-3.0 license. Comprehensive documentation describing the usage, installation, and configuration of the system can be found at https://bakta.readthedocs.io/en/latest.
